# Assessment of videos related to lung nodules in China

**DOI:** 10.3389/fsurg.2022.1019212

**Published:** 2022-10-10

**Authors:** Jiajun Han, Yifan Shi, Haitao Ma

**Affiliations:** ^1^ Department of Thoracic Surgery, The First Affiliated Hospital of Soochow University, Suzhou, China; ^2^ Department of Thoracic Surgery, Suzhou Dushu Lake Hospital, Suzhou, China

**Keywords:** lung nodule, health information, social media platform, quality, TikTok

## Abstract

**Background:**

With the popularization of mobile phones and the development of the Internet, many patients use social media platforms to seek health information. Currently, TikTok, iQiyi, Bilibili, and Weibo are the most popular video platforms in China. Therefore, based on the above facts, this study estimated the quality of lung nodule videos taken in China using these platforms.

**Methods:**

The term “lung nodule” was searched on these platforms. Then, the first 30 videos were selected. Subsequently, some videos were excluded after they had been reviewed and analyzed, after which information on the features and sources of these videos was finally assessed using DISCERN, the Journal of the American Medical Association (JAMA) Benchmark Criteria, and the Hexagonal Radar Schema. Analysis was performed according to different groups.

**Results:**

101 videos were included in this study. According to the different sources, although most videos were from physicians (71.3%), comprising those with shorter durations; faster updates; and more likes, comments, and shares; no significant difference in the scores were obtained. Moreover, regarding the different platforms, while Weibo had the highest update, TikTok had more likes, comments, and shares. Investigations also revealed that while score differences were recorded, most videos were rated “very poor” and “poor.” Besides, hexagonal radar charts showed a severe deficiency of video information.

**Conclusions:**

Although the quality of most videos on the understudied social media platforms was poor, these platforms have huge potential. Therefore, caution should be exercised when using the platforms as information sources about lung nodules, and a better review and push system is needed.

## Introduction

Lung cancer is a major global cancer type. A large number of patients with lung nodules were identified during the coronavirus disease-2019 outbreak, which enhanced physical examination awareness, resulting in a large panic. Therefore, with the popularity of mobile phones and the development of the Internet, social media platforms have become an essential way for people to obtain health-based information (1–3). However, differences in the ability and level of the uploaders, including the review and push systems of the platforms, have been considered to differ widely. Therefore, while the information people obtain is often incomplete or outdated, certain deviations have also been observed (3–6).

TikTok, iQiyi, Bilibili, and Weibo are the most popular social media platforms in China, each with hundreds of millions of active users. However, although there are studies that have compared and studied the videos of other specialties on some platforms (7–9), thus far, none exists about thoracic surgery. Therefore, this study chose lung nodules, a hot topic in thoracic surgery, as an entry point to evaluate the quality of videos related to thoracic surgery on these platforms.

## Methods

### Ethics statement

Since all videos on these social media platforms were publicly available, ethical approval was not needed.

### Video search strategy

The search was performed on social media platforms (i.e., TikTok, iQiyi, Bilibili, and Weibo) from July 19th to 21st, 2022. The search term was “肺结节” (lung nodule). Notably, newly registered accounts were used to reduce the impact on search results. Then, while the first 30 videos were reviewed and analyzed on each platform, irrelevant videos, commercial videos, videos without audio, and duplicate videos were excluded.

Subsequently, we analyzed each video according to the following features: duration, sources, number of days online, likes, comments, and shares. Sources were classified as physicians, news media, and independent users.

### Evaluating methodologies

During this study, DISCERN, the Journal of the American Medical Association (JAMA) Benchmark Criteria, and the Hexagonal Radar Schema were used for quality analyses of the videos (1, 9–11). We also estimated specialized medical issues related to thoracic surgery based on the National Comprehensive Cancer Network Guidelines of Non-small Cell Lung Cancer 2021.

Notably, DISCERN is a widely used instrument for assessing the quality of health information (12). It comprises 16 questions, with each question scored from one to five points. These 16 questions are then divided into three sections: reliability of the publication (questions 1–8), quality of the information about treatment choices (questions 9–15), and the overall score of the publication (question 16). Subsequently, we obtained a total DISCERN score by summing the scores from questions 1–15, after which all videos were divided into five groups based on their total DISCERN scores as follows: very poor (<27), poor (27–38), fair (39–50), good (51–62), and excellent (63–75) (13).

Contrastively, the JAMA benchmark criteria were used to evaluate the basic quality and reliability of the videos (14, 15). It comprised four items (authorship, attribution, disclosure, and currency), each of which was assigned one point.

However, the Hexagonal Radar Schema is a coded scale that reﬂects six dimensions of the video, including its definition, signs, risk factors, examinations, management, and outcomes (9, 16). According to the detailed criteria and examples, points from zero (not addressed at all) to two (fully addressed) were first given at each dimension, after which the spotlight and weight of each video could be visually presented by the shape and size of the radar chart. Any dimension scoring more than one point in this chart was considered acceptably clear.

Two experienced thoracic surgeons (JH and YS) evaluated all videos, and any disagreements were resolved by discussion with a third author (HM) for consensus.

### Statistical analysis

Statistical analyses were performed with IBM SPSS for Windows, version 19.0 (IBM Corp., Armonk, NY, United States). Categorical variables were presented as frequencies and ratios (%), whereas continuous variables were presented as the mean ± standard deviation (mean ± SD) and median (interquartile spacing) [M (IQR)]. Furthermore, the Kruskal‒Wallis test was used to determine statistically significant differences between more than two groups of any independent variable. Categorical data were compared using Fisher's exact probability test. *P*-value < 0.05 was considered significant.

## Results

**Figure 1:**
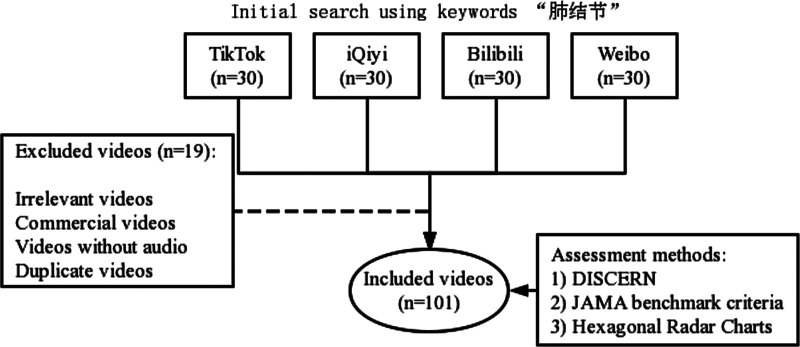
Flowchart of video selection.

**Figure 2:**
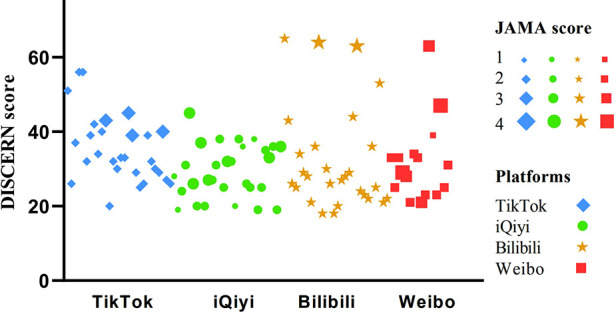
Detailed results of DISCERN and JAMA scores of the understudied video.

The term “lung nodule” was searched on these platforms (i.e., TikTok, iQiyi, Bilibili, and Weibo). Then, the first 30 videos were selected. After screening according to the exclusion criteria, 101 videos were included and analyzed (Figure 1). Investigations revealed that while the mean DISCERN total score for all videos was 32.05 ± 10.61 [median (IQR): 30 (12)], the mean JAMA score was 2.44 ± 0.65 [median (IQR): 2 (1)]. Figure 2 shows the detailed results of the DISCERN and JAMA scores of the understudied video. Detailed data on the general characteristics and scores of the understudied videos are shown in Table 1.

**Table 1:** General characteristics and scores of the understudied videos.

Category	mean ± SD	M (IQR)
Duration (s)	159.87 ± 292.90	107 (65)
Number of days online	326.25 ± 379.59	162 (393)
Number of likes	8,451.42 ± 23,690.85	64 (4,103)
Number of comments	442.16 ± 1,417.09	5 (230)
Number of shares	3,208.18 ± 11,787.56	41 (706)
DISCERN reliability	18.52 ± 5.33	18 (7)
DISCERN treatment	12.09 ± 5.88	10 (6)
DISCERN quality	2.74 ± 1.14	3 (2)
DISCERN total	32.05 ± 10.61	30 (12)
JAMA score	2.44 ± 0.65	2 (1)

Subsequently, the videos were grouped according to different sources, which revealed that most of the videos were uploaded by physicians (68.3%). The duration, number of days online, likes, comments, and shares were also significantly different (*P* < 0.05). Notably, videos uploaded by physicians had a poor quality; were shorter; more updated; and received more likes, comments, and shares. Nevertheless, no significant difference in the scores was observed among the three groups (*P* > 0.05). The specific values obtained are provided in Table 2.

**Table 2:** General characteristics and scores of the understudied videos from different sources.

Category	Physicians	News media	Independent users	*P*-value
Number of videos	69	26	6	-
Duration (s)	101.00 (74)	119.50 (60)	185 (815)	0.031
Number of days online	118 (245)	414 (1,095)	808 (1,090)	0.01
Number of likes	246 (12,973)	19.50 (64)	28.5 (64)	0.000
Number of comments	28 (507)	0 (1)	0 (3)	0.000
Number of shares	143 (1,174)	8.50 (24)	36 (38)	0.005
DISCERN reliability	18 (7)	16 (8)	18.5 (9)	0.172
DISCERN treatment	9 (6)	11 (6)	10.5 (6)	0.586
DISCERN quality	3 (1)	2.5 (2)	3.5 (2)	0.432
DISCERN total	29 (13)	30.5 (16)	31.5 (16)	0.673
JAMA score	2 (1)	3 (1)	2 (1)	0.442

The number of videos included and analyzed on TikTok, iQiyi, Bilibili, and Weibo was 28, 29, 28, and 16, respectively. We also observed that the number of days online, likes, comments, and shares significantly differed between the different platforms (*P* < 0.05). Specifically, while videos on Weibo had the highest update intensity, those on TikTok had more likes, comments, and shares. Moreover, from the DISCERN subitem and total score, although videos on TikTok had higher scores than on other platforms, the median scores on this platform were in the very poor or poor groups. However, with the JAMA score, videos on Bilibili were higher than on other platforms. The specific values obtained are provided in Table 3.

**Table 3:** General characteristics and scores of the understudied videos on the different platforms.

Category	TikTok	iQiyi	Bilibili	Weibo	*P-*value
Number of videos	28	29	28	16	-
Duration (s)	94.5 (79)	115 (58)	103 (62)	126.5 (102)	0.335
Number of days online	171.5 (223)	540 (885)	105 (265)	36 (79)	0.000
Number of likes	15,300 (30,104)	10 (35)	133.5 (400)	13.5 (25)	0.000
Number of comments	755 (1,700)	0 (1)	20 (52)	1 (4)	0.000
Number of shares	1,831.5 (10,820)	6 (17)	73 (164)	9 (26)	0.000
DISCERN reliability	21 (6)	17 (5)	16 (10)	19 (9)	0.000
DISCERN treatment	13 (9)	10 (5)	9 (5)	9 (5)	0.040
DISCERN quality	3 (1)	2 (2)	2 (1)	2 (1)	0.067
DISCERN total	33 (11)	28 (12)	27.5 (14)	28.5 (11)	0.048
JAMA score	2 (0)	2 (1)	3 (0)	2 (1)	0.000

According to the DISCERN classification data, investigations also revealed differences in duration, numbers of likes, comments, and shares. Specifically, we observed that while higher-quality videos appeared longer (*P* < 0.05), differences in the number of likes, comments, and shares had nothing to do with the quality of the video (*P* > 0.05). Furthermore, although no difference was observed between the sources of the DISCERN classification data (*P* > 0.05), differences were recorded between the different platforms (*P* < 0.05). The specific values obtained are provided in Table 4.

**Table 4:** DISCERN classification data of the understudied videos.

Category	Very poor	Poor	Fair	Good	Excellent	*P*-value
**Video characteristics, median (IQR)**						
Duration (s)	93 (72)	107 (44)	140 (126)	291 (413)	362 (1,960)	0.002
Number of days online	134 (303)	230 (654)	181 (416)	115 (305)	142 (451)	0.249
Number of likes	48 (229)	35 (3,664)	7,942 (36,452)	15,300 (29,058)	116.5 (779)	0.006
Number of comments	3 (26)	2 (115)	244 (1,879)	2,816.5 (10,035)	14.5 (348)	0.009
Number of shares	16 (128)	24 (675)	1,015 (11,460)	18,464.5 (80,638)	115.5 (691)	0.001
**Video source, *n***						0.199
Physicians	26	27	11	4	1	
News media	10	12	2	0	2	
Independent users	1	4	0	0	1	
**Platform, *n***						0.009
TikTok	5	12	8	3	0	
iQiyi	12	16	1	0	0	
Bilibili	13	9	2	1	3	
Weibo	7	6	2	0	1	

**Figure 3:**
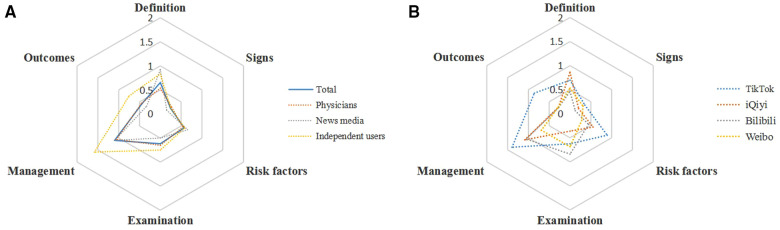
Hexagonal Radar Charts of videos. (**A**) Videos from different sources. (**B**) Videos on different platforms.

The hexagonal radar charts in Figure 3 showed that the average score of each dimension was less than one point, except for management, indicating a severe imbalance and inadequacy of video information. In addition, while no significant difference in scores was observed between the sources and platforms (*P* > 0.05), TikTok (53.6%) accounted for a higher proportion than other platforms, based on the number of videos that had full scores in one of the six dimensions. The specific values obtained are provided in Table 5.

**Table 5:** Hexagonal radar charts of the understudied videos.

Items	Definition	Signs	Risk factors	Examination	Management	Outcomes
Total	0.653	0.243	0.574	0.619	1.099	0.436
**Video source, *n***						
Physicians	0.536	0.275	0.543	0.652	1.058	0.449
News media	0.923	0.154	0.654	0.500	1.096	0.327
Independent users	0.833	0.250	0.583	0.750	1.583	0.750
**Platform, *n***						
TikTok	0.696	0.357	0.893	0.625	1.393	0.857
iQiyi	0.862	0.207	0.552	0.362	1.086	0.276
Bilibili	0.464	0.125	0.446	0.839	1.054	0.268
Weibo	0.531	0.313	0.281	0.688	0.688	0.281

## Discussion

Albeit inevitable, health problems are crucial concerns affecting people's lives daily and require immediate attention and accurate and prompt intervention. With the advancement of the Internet and the popularization of smartphones, social media platforms have become one of the popular venues for obtaining information. Because a strict review system is lacking (17), platforms are full of biased and outdated data, including misinformation. This will negatively affect health information dissemination and standardization of diagnosis and treatment (18–21). In thoracic surgery, lung nodules are currently one of the hotspots. An improper treatment orientation may delay the patient's treatment and lead to serious consequences. Therefore, an evaluation of videos on social media platforms in China from the perspective of thoracic surgeons should be conducted, which has not been investigated before.

The study found that in most videos, the DISCERN and JAMA scores were low. The low scores were primarily attributed to the lack of references and ownership. Although physicians had the highest percentage of uploads and were able to receive a good audience, the overall quality was poor. References often lacked descriptions, and further assessments could not be performed in these videos. Physicians were more inclined to describe the content in sections to ensure more attention. This was also one of the reasons why the Hexagonal Radar Schema and DISCERN scores were not high, and patients often receive incomplete information because of this. News media often use interviews in transmitting the information. After professional editing, the layers were often clearer; however, the time was often longer. With the accelerated pace of life, people often cannot perceive too much information in a short time. Studies have shown that long-term high attention reduces the audience's acceptance (22). Moreover, the platform lacked a standardized and professional review and comment system. The video's popularity mostly determined the strength of the push which had no absolute correlation with quality. As a result, high-quality videos fail to receive more attention.

The Hexagonal Radar Schema showed that in most dimensions, the information of each video was seriously insufficient, and an average score of more than 1 point was only achieved in the aspect of management. Because most patients with lung nodules lacked symptoms, very few videos involved descriptions of signs, which rendered the score to be low. Certainly, the content decomposition was also a major reason for this result. Concurrently, it could be found that more videos on TikTok have full marks in a single aspect; hence, the push of a series of videos may achieve a good effect.

Although it was impossible to directly count the viewing data of major platforms, the numbers of likes, comments, and shares on TikTok were far greater than that on the other platforms, indicating a higher popularity rate and larger audience (23, 24). Although the DISCERN subitem and total score were higher than those of other platforms, the average score was in the very poor or poor categories. Therefore, the overall quality needs to be improved. It was discovered that compared with other platforms, the search results of iQiyi were relatively fixed and the update was weak; the search results of Bilibili included a certain number of latest uploaded videos according to the search date, some of which had higher scores, which were always at the top of the search results, and due to the narrative of ownership, the JAMA score was higher than that recorded for other platforms. Weibo had the highest rate of irrelevant content and repetition, but it also had the highest update intensity. While there were differences in the video ratings across platforms, no platform demonstrated absolute dominance in the good and excellent categories.

Therefore, platforms should hire professionals to conduct more content reviews. To achieve adequate guidance, certain resources can be integrated and placed at the top of the search results. While ensuring the correctness of the content, uploaders, especially physicians, should try their best to highlight the guidelines or norms to make necessary checks.

This study has certain limitations. (1) All videos are in Chinese, and a comparative study of foreign videos should be conducted in subsequent research. (2) The retrieval results of all platforms change dynamically with the retrieval date. This is a cross-sectional study, and the analysis results of the collected data only represent the retrieval date. (3) Although a new account is created for information retrieval, different regions and retrieval times may yield different retrieval results depending on the platform algorithm. (4) All retrieval platforms only obtain the information of the first 30 videos in the results, and the data account for a small proportion. (5) Only the most popular media platforms in China were selected, so the results cannot reflect all media platforms. (6) Due to the lack of viewing data, it is impossible to evaluate the actual number of viewers, the actual viewing duration, and the actual viewing effect.

## Conclusions

In summary, social media platforms are a valuable tool for disseminating public health information. The existing videos related to lung nodules differ slightly between different sources and platforms, but their overall quality is poor, and some of them are incomplete or inaccurate. Therefore, establishing standardized review and reasonable push systems will play a significant role in disseminating public health information.

## Data Availability

The raw data supporting the conclusions of this article will be made available by the authors, without undue reservation.
